# Validation of the generalized anxiety disorder scales (GAD-7 and GAD-2) in primary care settings in Latvia

**DOI:** 10.3389/fpsyt.2022.972628

**Published:** 2022-10-06

**Authors:** Jelena Vrublevska, Lubova Renemane, Anda Kivite-Urtane, Elmars Rancans

**Affiliations:** ^1^Department of Psychiatry and Narcology, Riga Stradins University, Riga, Latvia; ^2^Institute of Public Health, Riga Stradins University, Riga, Latvia

**Keywords:** generalized anxiety disorder (GAD), mental disorder, primary care, validated anxiety screening, Mini International Neuropsychiatric Interview (MINI), Latvia, GAD-2, GAD-7

## Abstract

**Background:**

Anxiety disorders are the most prevalent mental disorders in the world and have an important impact on the global burden of disease. Generalized anxiety disorder (GAD) is the most prevalent anxiety disorder encountered in primary care. There are no available validated anxiety screening tools in primary care in Latvia. We aimed to validate both a seven-item and a two-item generalized anxiety disorder scale (GAD-7 and GAD-2) in the Latvian and Russian languages, to detect generalized anxiety disorder (GAD) in primary care settings in Latvia.

**Methods:**

During a 1-week period, all patients aged 18 years or older visiting their GP (general practitioners) with any health concern at 24 primary care settings throughout Latvia were invited to complete the GAD-7 in their native language (Latvian or Russian). Criterion validity was assessed against the Mini International Neuropsychiatric Interview (MINI).

**Results:**

The study sample included 1,459 participants who completed the GAD-7 and the MINI. The GAD-7 items showed good internal reliability [Cronbach's alpha 0.87 for Latvian version and 0.85 for Russian version (for Latvia) of the GAD-7]. A cut-off score for detecting GAD of 5 or above was estimated for Latvian version of the GAD-7 (sensitivity 75.4%, specificity 68.9%, respectively) and 7 or above for Russian version of the GAD-7 (sensitivity 73.3%, specificity 84.1%, respectively). The internal reliability of the GAD-2 was lower for both languages (Cronbach's alpha 0.75 for Latvian version and 0.68 for Russian version of the GAD-2). A cut-off score of 2 or above was established for both the Latvian, and Russian versions of the GAD-2 (sensitivity 78.9 and 83.3%; specificity 63.7 and 69.1% for the Latvian and Russian versions of the GAD-2, accordingly) for detecting GAD.

**Conclusions:**

This is the first study to report criterion validity of the Latvian and Russian (for Latvia) versions of the GAD-7 and GAD-2, assessed in a nationwide study conducted at the primary care level.

## Introduction

Anxiety disorders are the most prevalent mental disorders in the general population in the world and have a significant impact on the global burden of disease (1). They are receiving increasing attention because of their early onset as well as their tendency to recur and cause disability (2, 3). Estimates of the prevalence of anxiety disorders vary widely across studies and population groups. Different studies demonstrate lifetime prevalence rates of anxiety disorders ranging from 5.1 to 16.6% in general population, and from 7.2 to 19.5% in primary care (4–9). Moreover, anxiety disorders are often undetected, undertreated, and associated with the global health-related, personal and societal burden. In addition, they can cause substantial impairment of quality of life (10).

According to the latest evidence, anxiety disorders are becoming more prevalent. A recent systematic review estimated an additional 76.2 million cases of anxiety disorders globally (an increase of 25.6%). Additionally, the data suggest that anxiety disorders caused 44.5 million disability-adjusted life-years globally in 2020 (11). Another systematic review indicates that the rates of anxiety disorders in the general population could be more than 3 times higher in recent years (12).

Generalized anxiety disorder (GAD) is the most prevalent anxiety disorder encountered in primary care, with an estimated point prevalence of 8%. The disorder is present in 22% of primary care patients who complain of anxiety symptoms (9, 13). The high prevalence rates underline the necessity of identification and assessment of GAD in primary care settings, but many people who might benefit from treatment are not recognized. Moreover, of those patients who are diagnosed as suffering from GAD, 41% do not receive the adequate treatment (5). The data from previous studies suggest that GAD could be the most frequent anxiety disorder causing ‘completed' suicides; also sub-threshold GAD is clearly linked to suicide ideation (14). Anxiety disorders rank as the second leading diagnostic category (15.8%) in primary care in Latvia, based on the assessment with the MINI, with the prevalence of GAD of 6.1% (95% CI 4.9–7.3) (7).

It is estimated that the prevalence of diagnosis and treatment of anxiety disorders in primary care is much lower than expected, given their prevalence (15). The major problems in primary care are time constraints and the existence of comorbid depressive disorders and chronic physical health problems (16). Therefore, self-reported rating scales are often preferred in primary care level. The underdiagnosis of anxiety disorders appears to be a worrying issue for Latvia as well, since the data from the National Health Service Register show that the most prevalent diagnosed mental disorders in Latvia are organic mental disorders, schizophrenia spectrum disorders, but not neurotic and affective mental disorders, which are the most prevalent worldwide. Moreover, among neurotic spectrum disorders, Latvian GPs most frequently diagnose somatoform autonomic dysfunction (17, 18).

The NICE (National Institute for Health and Care Excellence) provides the evidence-based clinical guidelines for identification and assessment of common mental health problem, and recommends the use of the 2-item generalized anxiety disorder (GAD-2) tool for identification, and the 7-item generalized anxiety disorder scale (GAD-7) for assessment of anxiety disorder severity (19). A recent systematic review of validated screening tools for anxiety disorders that included 58 articles and 77 screening tools, demonstrated that the GAD-7 was one of the most commonly validated tools for anxiety disorders (20).

The GAD-7 was developed as a brief self-reported screening tool to detect probable cases of GAD among primary care patients, and assess its severity in clinical practice and research (21). The GAD-2, consists of the first two questions of the GAD-7, is a shorter version of the tool, and is used as a screening test for detection of GAD (5). The GAD-7 and the GAD-2 were validated in primary care patients and have been widely used by general practitioners (16). Earlier studies suggested that the GAD-7 and GAD-2 perform well for screening not only GAD, but can also be used for detecting other anxiety disorders such as panic disorder, social anxiety disorder and post-traumatic stress disorder (5, 16).

Till now, there are no published studies examining the psychometric properties of anxiety screening tools among the Latvian- and Russian-speaking population of Latvia. As the ethnic distribution of the Latvian population is more than 61% Latvian and the remaining are mostly Russian-speaking, it is critical to perform validation in both Latvian and Russian languages (22). Therefore, we aimed to investigate psychometric properties of the GAD-7 and GAD-2 to provide the reliability and validity of these tools, and recommended screening cut-off scores for GAD, using the Mini International Neuropsychiatric Interview (MINI) as the reference standard in a large sample among the Latvian primary care population.

## Materials and methods

### Procedure and participants

The study was conducted within the framework of the National Research Program, BIOMEDICINE 2014–2017, which aimed to estimate the prevalence of mental disorders in primary care settings in Latvia. The program was funded by the Latvian Ministry of Education and Science. The main aim of this program was to develop new methods and practices for the prevention, treatment and diagnosis of mental disorders, as also biomedical technologies to improve public health in Latvia. It comprised certain areas: cardiovascular and metabolic diseases, oncological diseases, and childhood and infectious diseases. Mental health was included in the program for the first time. Study participants did not receive any financial compensation for their participation. Within the project the validity of the PHQ-9 and the PHQ-2 was assessed and a cut-off score to identify depression was established (23). Patients visiting their general practitioners (GPs) for any medical reason were recruited from 24 primary care settings (16 in urban and 8 in rural regions) that covered all regions of Latvia. The survey was conducted in Latvian or in Russian, as per patient preference.

All patients, aged 18 years or older, visiting a primary care physician with any health concern, during a 1-week period, were invited to participate in the study. Those who visited their GPs for administrative reasons were not included. The others who were excluded were the patients who refused to participate in the study, patients younger than 18 years of age, and those who were not able to participate due to acute medical conditions requiring hospitalization or other general medical conditions (one patient was deaf-mute). All consecutive patients were invited to complete the paper-and-pencil form of the GAD-7 in their preferred language (Latvian or Russian) before seeing the GP, and were requested to complete a structured socio-demographic questionnaire. All ambiguities and questions that arose were clarified by the researcher.

The Mini International Neuropsychiatric Interview (MINI) Version 6.0.0 was conducted over the phone by four trained psychiatrists (who were unaware of the GAD-7 scores), no more than 2 weeks after the first contact with the patient. The MINI was used as the standard to determine the presence of GAD and other anxiety disorders. Participants with high scores of the GAD-7, the PHQ-9 and those who were diagnosed with GAD, or any other diagnostic category according to the MINI, were referred for appropriate care.

Riga Stradins University Ethics Committee approved this study (No. 8/18.06.2015.), and written informed consent was obtained from all participants. The study was carried out in accordance of the Declaration of Helsinki and its subsequent amendments.

### Measures

The GAD-7 consists of 7 self-reported items, measuring symptoms of anxiety, allowing the rapid screening for GAD. Each item has a Likert-response format on a 4-point scale (0–3 points). Respondents were asked to consider the previous 2 weeks and to rate symptom frequency as ‘not at all' (0), ‘several days' (1), ‘more than half of all days' (2) or ‘nearly all days' (3). The total score response ranged from 0 to 21. In the initial validation study of the GAD-7, estimated sensitivity and specificity were identified at 89 and 82%, respectively, at a cut-off score of 9 (21).

The GAD-2 is a shorter version of the tool that is composed of the first two questions of the GAD-7. The GAD-2 in its initial validation study had a sensitivity of 86% and a specificity of 83% at a cut-off score of 2 (5).

A forward/backward translation of the GAD-7 into the Latvian and Russian languages was performed by professional translators and was reviewed by Latvian and Russian language speaking psychiatrists. Additionally, the evaluation of potential problems in comprehension or cultural differences of scale was discussed in a professional focus group. The final agreement of both language versions of the GAD-7 was reached.

The MINI is a structured diagnostic interview for psychiatric disorders according to the Diagnostic and Statistical Manual of Mental Disorders, and the International Classification of Disease, 10th revision (24). It is widely used for research purposes in psychiatric and general populations, including primary care patients (25, 26). The MINI has been translated and adapted by authorship holders for use in 67 languages, including Latvian and Russian (27). It consists of 120 questions and screens 17 axis I disorders for 24 current and lifetime diagnoses. The interview was conducted over the telephone, which is acceptable and has been used in other studies (28). We administrated all modules of the MINI to identify current diagnoses of anxiety disorders, such as panic disorder, agoraphobia, social phobia, obsessive-compulsive disorder, posttraumatic stress disorder, and generalized anxiety disorder.

The participants' sex (male or female), age (18–34, 35–49, 50–64, or 65+ years), marital status (married/cohabiting, single, or living separately/divorced/widowed), employment status (employed, unemployed, or economically inactive), educational level (higher/unfinished higher education, general/vocational secondary/unfinished secondary education, or 9-year basic/unfinished basic education), and place of residence [urban: capital (Riga)/other city, or rural] were recorded.

### Statistical analysis

The internal consistency of the GAD-7 and GAD-2 was assessed by Cronbach's alpha coefficient, while their criterion validity was assessed by receiver operating characteristic (ROC) analysis. The criterion validity was analyzed in terms of sensitivity (true positive), specificity (true negative), positive and negative predictive values [PPV, NPV; the probability that individuals with a positive (negative) test result truly have (do not have) the condition], a positive likelihood ratio (LR+; “probability that a positive test would be expected in a patient divided by the probability that a positive test would be expected in a patient without a disease”), and a negative likelihood ratio (LR–; “the probability of a patient testing negative who has a disease divided by the probability of a patient testing negative who does not have a disease”) for different cut-off scores (29). The Latvian and Russian versions of the MINI, which were used to diagnose GAD and other anxiety disorders, served as the criterion standard. Data analyses were performed using IBM- SPSS (Statistical Package for the Social Sciences), version 26.0. A separate analysis was conducted for the responders who answered the survey in Latvian, and those who used Russian translation of the survey. ROC curves were created for each instrument. The area under the curve (AUC) which is a measure that provides an overall summary of the utility of the scale to correctly identify GAD cases was determined. The statistical significance of the differences of demographic characteristics between groups of mental disorders was assessed using Chi-Squared test of Fisher's exact test. The results were considered as statistically significant if *p* < 0.05.

## Results

Of the 1,756 patients who visited their GP, 152 refused to participate. At baseline, a sample of 1,604 patients was approached to complete the GAD-7 and GAD-2. Response rate among the patients was 91.3% and varied in the range 86.3–93.7% across 24 primary care settings all over the country. The questionnaires were completed by 1,585 participants. Of those who completed the screening questionnaire, 100 did not agreed to be interviewed with the MINI over phone or did not answer the telephone call three times within 2 weeks, and were excluded from the study. Those patients who were excluded from the study did not show statistically significant differences in sociodemographic status compared to those who were included. The remaining 1,485 patients were interviewed with the MINI over the telephone. The questionnaires of 18 patients had to be discarded due to insufficient data quality. Of the 1,467 patients, eight patients were missing because the language in which the GAD-7 was completed was not specified. Finally, 1,459 patients were included in the analysis.

The demographic characteristics of our study sample with respect to current anxiety disorders determined by the MINI are summarized in Table 1. According to the MINI, 61 patients (4.2%) were diagnosed with GAD, 142 patients (9.7%) with anxiety disorder without GAD and 28 patients (1.9%) had comorbidity of GAD and other anxiety disorders.

**Table 1:** Demographic characteristics of study sample with respect to current mental disorders established by the Mini International Neuropsychiatric Interview (*n* = 1,467).

**Variable**	**Total Sample**		**No anxiety disorders**		**GAD only**		**Anxiety disorders without GAD**		**GAD** + **other Anxiety Disorders**		***p***
	***n***	**%**	***n***	**%**	***n***	**%**	***n***	**%**	***n***	**%**	
**Total**	**1467**	**100.0**	**1236**	**84.3**	**61**	**4.2**	**142**	**9.7**	**28**	**1.9**	
**Sex**
Female	1019	69.5	839	67.9	43	70.5	113	79.6	24	85.7	**0.008**
Male	448	30.5	397	32.1	18	29.5	29	20.4	4	14.3	
**Age**
18–34	209	14.2	172	13.9	7	11.5	23	16.2	7	25.0	0.25
35–54	455	31.0	385	31.1	13	21.3	48	33.8	9	32.1	
55–64	349	23.8	288	23.3	19	31.1	34	23.9	8	28.6	
65+	454	30.9	391	31.6	22	36.1	37	26.1	4	14.3	
**Education**
Higher and unfinished higher	436	29.9	389	31.6	11	18.6	25	17.6	11	40.7	**0.001**
education					
General or vocational secondary	838	57.4	692	56.2	34	57.6	97	68.3	15	55.6	
and unfinished secondary					
9-year basic, unfinished basic	185	12.7	150	12.2	14	23.7	20	14.1	1	3.7	
**Employment status**
Employed	776	53.2	655	53.2	26	43.3	79	55.6	16	59.3	0.06
Unemployed	82	5.6	62	5.0	4	6.7	12	8.5	4	14.8	
Economically inactive	602	41.2	514	41.8	30	50.0	51	35.9	7	25.9	
**Marital status**
Married. cohabiting	895	61.3	768	62.4	33	55.0	81	57.0	13	48.1	0.09
Single	144	9.9	116	9.4	6	10.0	15	10.6	7	25.9	
Live separately, divorced, widowed	421	28.8	347	28.2	21	35.0	46	32.4	7	25.9	
**Place of residence**
Capital (Riga)	303	20.7	255	20.6	20	32.8	21	14.8	7	25.0	**0.005**
Other city	692	47.2	598	48.4	26	42.6	59	41.5	9	32.1	
Rural	472	32.2	383	31.0	15	24.6	62	43.7	12	42.9	

In the total sample (*n* = 1,467) the mean score of the GAD-7 was 4.1 [standard deviation (SD) 4.0] and of the GAD-2–1.5 (SD 1.4). Whereas in the group of patients with GAD as per the MINI (*n* = 89) the mean score of the GAD-7 and GAD-2 was 8.7 (SD = 5.1) and 3.0 (SD = 1.8), respectively.

Cronbach's alpha for the Latvian version of the GAD-7 and GAD-2 was 0.87 and 0.75, respectively, and for the Russian version of the GAD-7 was 0.85, indicating good internal consistency. However, Cronbach's alpha for the Russian version of the GAD-2 was found to be 0.68, demonstrating a questionable level of internal consistency.

All items in the GAD-7 for both languages were significantly and positively associated with the total GAD-7 scores, and Cronbach's alpha did not decrease if the items were deleted. The data presented in Tables 2, 3 demonstrate corrected item-total correlations, Cronbach's alpha, scale mean, and scale variance when an item is deleted from the GAD-7 scale in Latvian and Russian versions.

**Table 2:** Corrected item-total correlations and Cronbach's alpha, scale mean, scale variance when an item is deleted from the GAD-7 and GAD-7 in Latvian (*n* = 908).

	**Scale mean if an item deleted**	**Scale variance if an item deleted**	**Corrected item-total correlation**	**Cronbach's alpha if an item deleted**
**GAD-7 Latvian**
GAD7: 1. Feeling nervous. anxious or on edge	3.09	10.66	0.71	0.84
GAD7: 2. Not being able to stop or control worrying	3.63	11.07	0.74	0.84
GAD7: 3. Worrying too much about different things	3.31	10.78	0.68	0.85
GAD7: 4. Trouble relaxing	3.60	11.15	0.68	0.85
GAD7: 5. Being so restless that it is hard to sit still	3.80	12.28	0.60	0.86
GAD7: 6. Becoming easily annoyed or irritable	3.46	11.87	0.56	0.86
GAD7: 7. Feeling afraid as if something awful might happen	3.68	12.02	0.57	0.86
**GAD-2 Latvian**
GAD7: 1. Feeling nervous. anxious or on edge	0.46	0.53	0.60	.
GAD7: 2. Not being able to stop or control worrying	1.00	0.69	0.60	.

**Table 3:** Corrected item-total correlations and Cronbach's alpha, scale mean, scale variance when an item is deleted from the GAD-7 in Russian (*n* = 551).

	**Scale mean if item deleted**	**Scale variance if item deleted**	**Corrected item-total correlation**	**Cronbach's alpha if item deleted**
**GAD-7 Russian**
GAD7: 1. Feeling nervous, anxious or on edge	3.03	11.77	0.63	0.83
GAD7: 2. Not being able to stop or control worrying	3.72	12.45	0.69	0.82
GAD7: 3. Worrying too much about different things	3.36	11.54	0.66	0.82
GAD7: 4. Trouble relaxing	3.58	11.98	0.67	0.82
GAD7: 5. Being so restless that it is hard to sit still	3.85	13.69	0.55	0.84
GAD7: 6. Becoming easily annoyed or irritable	3.30	12.33	0.51	0.85
GAD7: 7. Feeling afraid as if something awful might happen	3.68	12.68	0.61	0.83
**GAD-2 Russian**
GAD7: 1. Feeling nervous. anxious or on edge	0.37	0.50	0.52	.
GAD7: 2. Not being able to stop or control worrying	1.06	0.79	0.52	.

**Figure 1:**
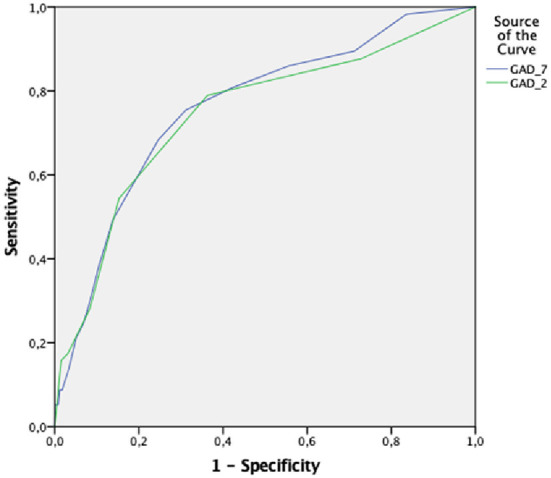
ROC (reciever operating characterstics curve of GAD-7 and GAD-2 in Latvian.

**Figure 2:**
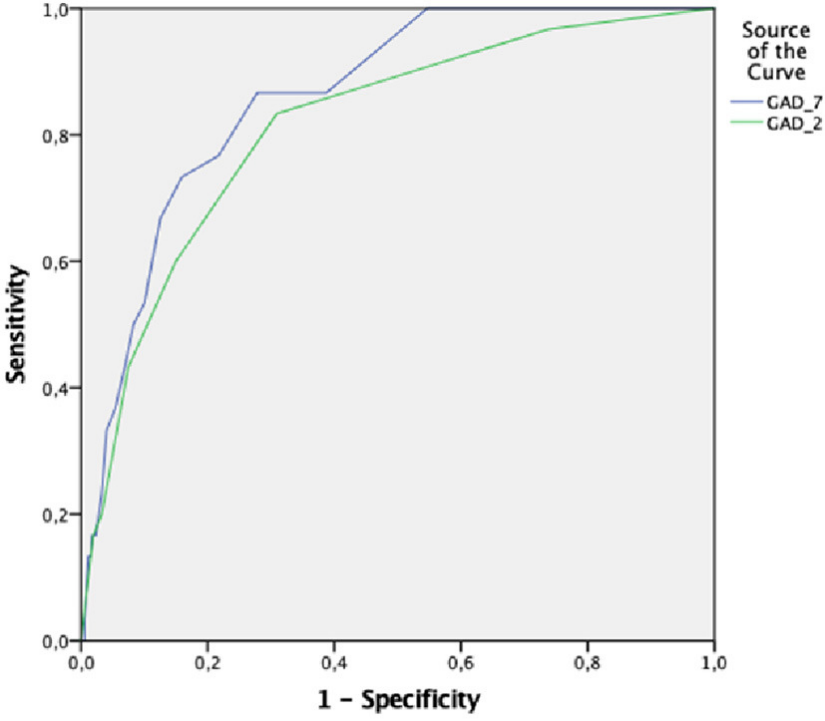
ROC (reciever operating characterstics curve of GAD-7 and GAD-2 in Russian.

The ROC analysis of the GAD-7 and GAD-2 for the diagnosis of GAD, established by the MINI, is shown in Table 4. The ROC curves of the GAD-7 and GAD-2 are illustrated in Figure 1 for Latvian versions and in Figure 2 for Russian versions.

**Table 4:** The ROC analyses of the GAD-7 and GAD-2 Latvian and Russian versions for the diagnosis of GAD established by the MINI (*n* GAD-7 and GAD-2 Latvian = 908; *n* GAD-7 and GAD-2 Russian = 551).

**Cut of score**	**Sensitivity, %**	**Specificity, %**	**PPV, %**	**NPV, %**	**LR+**	**LR–**
**LATVIAN**
**GAD-7**
≥3	86.0	44.4	9.4	97.9	1.55	0.32
≥4	80.7	57.9	11.4	97.8	1.92	0.33
≥5	75.4	68.9	14.0	97.7	2.42	0.36
≥6	68.4	75.3	15.7	97.3	2.77	0.42
≥7	57.9	81.3	17.2	96.6	3.10	0.52
≥8	49.1	86.3	19.3	96.2	3.58	0.59
≥9	38.6	89.4	19.6	95.6	3.64	0.69
≥10	29.8	91.7	19.3	95.1	3.59	0.77
≥11	24.6	93.2	19.4	94.9	3.62	0.81
≥12	21.1	94.9	21.8	94.7	4.14	0.83
≥13	17.5	95.8	21.7	94.5	4.17	0.86
≥14	14.0	96.6	21.6	94.4	4.12	0.89
≥15	8.8	98.2	25.0	94.1	4.89	0.93
**GAD-2**
≥1	87.7	27.0	7.5	97.0	1.20	0.46
≥2	78.9	63.7	12.7	97.8	2.17	0.33
≥3	54.4	84.7	19.3	96.5	3.56	0.54
≥4	28.1	91.7	18.4	95.0	3.39	0.78
≥5	17.5	96.8	27.0	94.6	5.47	0.85
≥6	15.8	98.5	40.9	94.6	10.53	0.85
**RUSSIAN GAD-7**
≥3	100.0	45.3	9.5	100.0	1.83	0.00
≥4	86.7	61.2	11.4	98.8	2.23	0.22
≥5	86.7	72.2	15.2	98.9	3.12	0.18
≥6	76.7	78.3	16.9	98.3	3.53	0.30
≥7	73.3	84.1	21.0	98.2	4.61	0.32
≥8	66.7	87.5	23.5	97.9	5.34	0.38
≥9	53.3	90.0	23.5	97.1	5.33	0.52
≥10	50.0	91.7	25.9	97.0	6.02	0.55
≥11	43.3	93.1	26.5	96.6	6.28	0.61
≥12	36.7	94.6	28.2	96.3	6.80	0.67
≥13	33.3	96.0	32.3	96.2	8.33	0.69
≥14	23.3	96.7	29.2	95.6	7.06	0.79
≥15	16.7	97.7	29.4	95.3	7.26	0.85
**GAD-2**
≥1	96.7	26.3	7.0	99.3	1.31	0.13
≥2	83.3	69.1	13.4	98.6	2.70	0.24
≥3	60.0	85.0	18.8	97.4	4.00	0.47
≥4	43.3	92.5	25.0	96.6	5.77	0.61
≥5	20.0	96.7	26.1	95.5	6.06	0.83
≥6	16.7	98.1	33.3	95.3	8.79	0.85

PPV, positive predictive value; NPV, negative predictive value; LR+, positive likelihood ratio; LR–, negative likelihood ratio.

The ROC analysis of the GAD-7 in Latvian exhibited an area under the curve (AUC) of 0.76 (SE = 0.03; *p* = 0.000; 95% CI = 0.70–0.83). Youden's index was highest with a cut-off score of 5 or above, and the GAD-7 sensitivity was 75.4%, specificity was 68.9% with a PPV of 14.0% and a NPV of 97.7%, a LR+ of 2.4 and a LR– of 0.36 for this cut-off score.

The ROC analysis of the GAD-2 in Latvian exhibited the AUC of 0.74 (SE = 0.04; *p* = 0.000; 95% CI = 0.67–0.82), and Youden's index was highest with a cut-off score of 2 or above (Figure 1). At this cut-off score the GAD-2 sensitivity was 78.9% and specificity was 63.7%, with the PPV (positive predictive value) of 12.7% and the NPV (negative predictive value) of 97.8, and the LR+ of 2.17 and the LR– of 0.33.

For the Russian version of the GAD-7 and GAD-2, the AUC (area under the ROC curve) in the ROC analysis was 0.86 (SE = 0.03; *p* = 0.000; 95% CI = 0.81–0.92) and 0.81 (SE = 0.04; *p* = 0.000; 95% CI = 0.74–0.89), respectively (Figure 2).

A cut-off score of 7 or above for the GAD-7 Russian language demonstrated sensitivity of 73.3% and specificity of 84.1%, with the PPV of 21.0% and the NPV of 98.2%, and the LR+ of 3.53 and the LR– of 0.30.

The GAD-2 Russian version indicate sensitivity of 83.3% and specificity of 69.1% at a cut-off score 2 or above. The PPV was 13.4% and the NPV was 98.6, and the LR+ was 4.61 and the LR– was 0.32 at this cut-off score.

## Discussion

The present study aimed to investigate the validity of the GAD-7 and GAD-2 Latvian and Russian versions, for Latvia, and to identify a cut-off score to detect the symptoms of GAD in a nationwide sample of patients who visited their GP due to any medical reason. The reference standard in our study was a structured clinical interview (MINI) that was conducted by four trained psychiatrists. This screener so far is the only questionnaire that has been tested for anxiety symptoms in a primary care in Latvia.

Validation of the GAD-7 and GAD-2 scales has earlier been carried out in different settings, languages and populations worldwide, for example, among pregnant women, among patients with migraine, HIV, and epilepsy, and among high school students, indicating that these tools are valid and useful for screening GAD (21, 30–35).

The initial validation study for the GAD-7 that was performed in 15 primary care clinics in the United States, had a Cronbach's alpha of 0.92, and at a cut of score of 9, the GAD-7 had a sensitivity of 89%, and specificity of 82% (21).

In terms of reliability, the GAD-7 and GAD-2 Latvian versions and GAD-7 Russian version had good internal consistency (Cronbach's alpha of 0.87 for the GAD-7, 0.75 for the GAD-2 Latvian version, and 0.85 for the Russian version of the GAD-7). This result supports the homogeneity of the scale and the contribution of all the items to the measurement of anxiety symptoms. However, the Russian version of the GAD-2 had demonstrated lower level of internal consistency (Cronbach's alpha of 0.68) in comparison with the Latvian version.

Our study showed that at a cut-off score of 5 or above for the GAD-7-Latvian version, and at a cut- off score of 7 or over for the GAD-7-Russian version, had the highest sum of specificity and sensitivity. A recent systematic review of validated screening tools for anxiety disorders in low to middle income countries identified six validation studies of the GAD-7 that were performed in different population groups with a similar methodological approach. In this review, a wide range of sensitivity (57–94%) and specificity (53–94%) was reported at cut-off scores 6 to 10, that varied depending on the regions where the studies were conducted, and sample size (20).

In another systematic review and diagnostic meta-analysis that aimed to evaluate the accuracy of the GAD-7 and GAD-2 questionnaires to identify anxiety disorders, 12 samples with 5,223 participants were analyzed. The authors suggested that the GAD-7 had acceptable properties for identifying GAD at cut-off scores ranging from 7 to 10 (36).

In a Finnish validation study of the GAD-7 carried out in primary care, it was found that the sensitivity and specificity for GAD with a cut-off point of 7 or more were 100.0 and 82.6%, respectively (34).

The identified cut-off score for the GAD in Latvian language was lower in comparison with previous studies carried out in primary care, however, the score for Russian version was consistent with a Finnish validation study of GAD-7 (34). Identified differences in the cut-off points of the GAD-7 across the studies support the suggestion that specific validation of scales is required for each country, population group and language.

The literature data on validation of the GAD-2 in primary care are limited, since it has not been as frequently validated as the GAD-7. The first validation study of the GAD-2 was done in 2007 on the primary care population of the United States of America, in which reported sensitivity and specificity were 86 and 83%, respectively, at a cut of score of 3 or greater (5). The systematic review and meta-analysis carried out in 2016, identified six samples that provided data on the accuracy of the GAD-2 for detecting GAD. The meta-analysis data suggested that pooled sensitivity and specificity values appeared acceptable at a cut-off point of 3 [sensitivity: 0.76 (95% CI 0.55–0.89), specificity: 0.81 (95% CI 0.60–0.92)] (36). The validation of a Finnish translation of the GAD-7 and GAD-2 screening tools in primary care population indicated a sensitivity of 0.83 and specificity of 0.90, at a cut-off point of 3 or more for the GAD-2 (34). Our study demonstrated that a cut-off score of 2 in the GAD-2 for both languages has the best sensitivity and specificity, and it was lower than in previous studies (5, 34, 36). Notably, the validation study of the GAD-7 and GAD-2 in patients with migraine demonstrated a cut-off score of 5 for the GAD-7, with sensitivity of 78.1% and specificity of 74.6% for the GAD-7, and a cut-off score of 1 for the GAD-2, with sensitivity of 44.6% and specificity of 94.3%, which is lower than in our study (35). These findings once again underline the necessity to validate scales in specific population groups and local languages.

Differences in cut-off scores across the countries can be explained with respect to study's settings, specific disease groups, sample size and characteristics (34, 37, 38). Another explanation includes cultural and language based differences in expression of psychopathology, and different interpretations of grading using the Likert scale (35, 39). Vast amount of literature is highlight the need for culturally and ethnically sensitive GAD screening tools (40).

Our data demonstrate that the Latvian and Russian (for Latvia) translations of the GAD-7 and GAD-2 are valid screening tools with acceptable sensitivity and specificity for GAD. Additional information is needed to further define the optimal cut-off point for Latvian and Russian versions. The GAD-7 and GAD-2 could be validated for other anxiety disorders in the future, as has been done in previous studies (5, 34).

The strengths of this study include the fact that all patients were from primary care and all of them received a MINI assessment as the reference standard. Our study included a large sample size of patients in primary care, which covered all regions of Latvia and was conducted in urban as well as rural areas. Moreover, in the study, only those patients were included, who visited their GP due to medical reasons. The respondents were assessed in the language of their preference. The patients were interviewed by four trained psychiatrists who were unaware of the GAD-7 estimates. Finally, GAD cases without any other comorbid mental disorders were included in the data analysis. Further studies in other clinical populations are necessary to evaluate its sensitivity and specificity as well as cut-off points to screen for GAD and other anxiety disorders.

This study has important practical implications. In early 2021, in response to the negative impact of the Covid-19 pandemic on the mental health of the population, the Ministry of Health of Latvia issued an information report on the dynamic follow-up of patients with mental and behavioral disorders conducted by GPs, that is, “Dynamic observation of patients with mental and behavioral disorders by a family doctor” (41). The Ministry of Health, together with mental health professionals and GPs, has developed easy-to-read algorithms using our validated GAD-2 and GAD-7 scales to help the GP assess patients with mental health issues, in order to make a diagnosis and select the appropriate treatment path and specialists to be consulted. Patients with prevalent anxiety disorders, for whom the GP does not consider referral to be necessary, can be adequately treated at the primary care level. Implementation of the GAD at the primary care level might contribute to improvement in recognition of anxiety spectrum disorders.

## Conclusion

In summary, the Latvian and Russian versions of the GAD-7 and GAD-2 have moderate psychometric properties for screening for GAD. The optimal cut-off score of the GAD-7 Latvian and Russian version for Latvia, which had the best psychometric characteristics for detecting GAD, was 5 or above and 7 or above, accordingly. The recommended cut-off score of the GAD-2 was 2 or above for both Latvian and Russian versions.

There are several limitations in our study. First, there was a rather small sample size of GAD cases according to the MINI. Meanwhile, small sample size might reflect the differences in sensitivity and specificity compared with other studies. Second, the data demonstrated the prevalence of anxiety disorders and validity of the GAD-7 and GAD-2 for determining of GAD in a primary care population, which eliminates the potential to characterize individuals and use the GAD-7 and GAD-2 observed in specialized psychiatric outpatient departments, clinical settings, and the general population. However, our target population involved persons visiting primary care settings. The GAD-7 and GAD-2 consist of a self-report questionnaire. These screening instruments only provide a probable diagnosis of GAD that has to be investigated by further evaluation. Another limitation of our study is meaning of the LR; at a cut-off point of 5 or over for the GAD-7 Latvian version, LR+ of 2.42 and the LR– of 0.36 were found; in the Russian version, at a cut-off point of 7 or higher LR+ of 4.61 and LR– of 0.32 were found. The GAD-2 Latvian version, at a cut-off point of 2 or over, demonstrated LR of 2.17+ and LR– of 0.33, and the Russian version, at a cut-off point of 2 or over, had the LR+ of 2.70 and LR– of 0.24. These rates of the LR reflect rather small probability and sometimes useful test levels for all versions of the scales. The GAD-7 measures anxiety over the past 2 weeks, however, the MINI measures the GAD over the past 6 months. The difference in the observation period between the two instruments may affect probability of the usefulness of the GAD-7 and GAD-2. Additionally, one of the limitations is cross-sectional design of the study; there is a need for larger number of patients with GAD to improve the statistical significance of our findings, longitudinal studies are needed to establish the sensitivity to change. Future research should consider exploring psychometric properties using exploratory factor analysis and confirmatory factor analysis of the GAD-7 and the GAD-2 Latvian and Russian versions. Inclusion of currently diagnosed and treated patients may increase bias by inflating estimates of screening accuracy.

## Data availability statement

The raw data supporting the conclusions of this article will be made available by the authors, without undue reservation.

## Ethics statement

The studies involving human participants were reviewed and approved by Ethics Committee of Research in Riga Stradins University. The patients/participants provided their written informed consent to participate in this study.

## Author contributions

JV, LR, AK-U, and ER conceived the presented idea and study design and analyzed the data. AK-U was responsible for the statistical data analysis. JV and LR wrote the first version of the manuscript. All authors participated in interpreting the data and developing further stages and the final version of the paper. All authors contributed to the article and approved the submitted version.

## Funding

The study was supported by The National Research Programme BIOMEDICINE 2014–2017 (Nr. 5.8.1.).

## Conflict of interest

The authors declare that the research was conducted in the absence of any commercial or financial relationships that could be construed as a potential conflict of interest.

## Publisher's note

All claims expressed in this article are solely those of the authors and do not necessarily represent those of their affiliated organizations, or those of the publisher, the editors and the reviewers. Any product that may be evaluated in this article, or claim that may be made by its manufacturer, is not guaranteed or endorsed by the publisher.
